# Personalised proteome analysis by means of protein microarrays made from individual patient samples

**DOI:** 10.1038/srep39756

**Published:** 2017-01-03

**Authors:** Syafrizayanti, Smiths S. Lueong, Cuixia Di, Jonas V. Schaefer, Andreas Plückthun, Jörg D. Hoheisel

**Affiliations:** 1 Division of Functional Genome Analysis, Deutsches Krebsforschungszentrum (DKFZ), Im Neuenheimer Feld 580, 69120 Heidelberg, Germany; 2 Department of Chemistry, Faculty of Mathematics and Natural Sciences, Andalas University, Kampus Limau Manis, Padang, Indonesia; 3 Key Laboratory of Heavy Ion Radiation Medicine of Gansu Province, Institution of Modern Physics, Chinese Academy of Science, 509 Nanchang Road, 730000 Lanzhou, China; 4 Department of Biochemistry, University of Zürich, Winterthurerstraße 190, 8057 Zürich, Switzerland

## Abstract

DNA sequencing has advanced to a state that permits studying the genomes of individual patients as nearly a matter of routine. Towards analysing a tissue’s protein content in a similar manner, we established a method for the production of microarrays that represent full-length proteins as they are encoded in individual specimens, exhibiting the particular variations, such as mutations or splice variations, present in these samples. From total RNA isolates, each transcript is copied to a specific location on the array by an on-chip polymerase elongation reaction, followed by *in situ* cell-free transcription and translation. These microarrays permit parallel analyses of variations in protein structure and interaction that are specific to particular samples.

A comprehensive DNA sequence analysis of patient genomes will relatively soon become clinical routine and provide a powerful tool for personalised medicine. However, many pathological mechanisms that underlie disease are caused by biochemical activities or signal transduction processes that are mediated by proteins. Also, the large majority of therapeutic modalities target proteins. First draft maps of the human proteome were published^1^,^2^,^3^. For a personalised view, however, it is crucial to know about protein variants that are expressed in individual patients, caused by mutations or differential splicing for example, since they directly influence protein activity and the interaction with other cellular components or drugs^4^. The naturally occurring protein variations encoded by the about 22,000 human genes are estimated to number up to a million. This is augmented by an unknown number of disease-relevant variations. How variations influence the affected proteins can rarely be predicted from DNA sequence^4^. Here, we report a procedure that allows isolating and making available to interaction studies and other diagnostic processes the proteins as they are present in a particular sample of an individual person.

Since dealing with many molecules simultaneously, protein microarrays provide a suitable assay format to such ends. Many proteins are accessible at a time and analysis is based on their natural, inherent affinity properties. One way to produce the arrays is by spotting proteins, which are expressed in recombinant cells, isolated and purified prior to array production^5^,^6^. For analysing many proteins of an individual patient, however, such an approach – requiring cloning, expression and purification for all proteins of interest – is extremely laborious. Alternatively, protein microarrays can be produced by cell-free expression from DNA-templates, utilising cell lysates that contain the entire machinery for *in vitro* transcription and translation^7^. Different formats exist, such as the original ‘protein *in situ* array’ (PISA)^8^, the ‘nucleic acid programmable protein array’ (NAPPA)^9^, the ‘DNA array to protein array’ (DAPA)^10^ or production by the ‘multiple spotting technique’ (MIST)^11^. In all formats, gene sequences are amplified by a polymerase chain reaction (PCR), introducing during the process functional features, such as promoter and ribosomal binding site (RBS); also, epitope tags or other experimentally useful structures can be added. The PCR-products are spotted onto the arrays and form the templates for *in situ* expression. While this avoids the bottlenecks of gene cloning, cell growth and protein purification, the DNA templates are still generated in separate reactions and need to be transferred to the microarray prior to protein expression. Our method merges all the steps on the array, thereby enabling preparation of individualised protein arrays, but avoiding individual handling.

## Results

### On-chip polymerase elongation reaction

**Figure 1:**
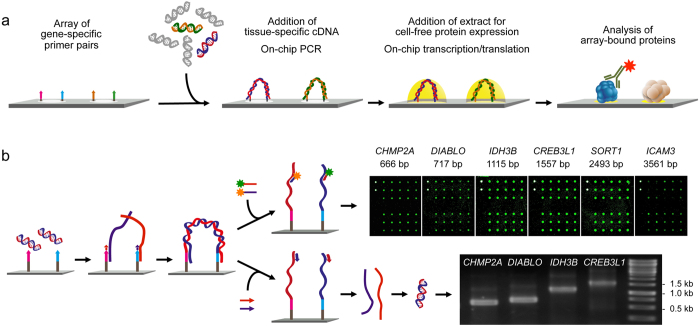
Principle and performance of the process of producing personalised protein microarrays. (**a**) Schematic illustration of the overall process. (**b**) Quality assessment of the on-chip PCR. For direct detection, labelled oligonucleotides were hybridised that were complementary to the newly synthesised DNA strands (top). Alternatively, appropriate primers were added; after a polymerase extension reaction, the single-stranded DNA products were eluted, PCR-amplified and characterised by gel electrophoresis (bottom).

For all genes of interest, a flanking pair of primers is present at defined positions on the surface (Fig. 1a). Upon incubation with total cDNA from an individual sample, on-chip polymerase elongation is performed, producing exact DNA copies of the gene transcripts with all their individual variations, such as mutations or differential splicing. This polymerase reaction differs markedly from normal PCR. In normal PCR, the primers are present in abundance. Also, there is a reverse primer binding to the newly synthesised DNA. On the microarray, the only primer molecules present are the ones attached to the surface. Each of them gets extended and there is no inverse primer for amplification. Therefore, there cannot be any out-competition of molecules. In case of two isoforms being present, for example, the ratio, by which they are represented eventually, is defined by the ratio, by which the two isoforms are present in the mixture; also their length does not matter (Supplementary Fig. 1). Hybridisation and annealing are controlled by kinetics and mass transport and thus the ratio of abundance^12^,^13^. Temperature cycling is only done in order to hybridise template also to primers that were not extended during the initial elongation cycle.

From these DNA copies, full-length proteins are produced by *in situ* transcription and translation, which resemble the proteins actually present in the original sample. The immobilised primers used (see Methods for details) encode a T7 promoter and ribosomal binding site (RBS). Additionally, they contain sequences for an N-terminal 6×His and C-terminal V5 tag, which allows an antibody-based detection of the eventual proteins. Apart from gene-specific primers, also oligonucleotides were used that permit the copying of genes from cDNA clones via vector sequences. As the thermostable attachment of the primers to the microarray surface was achieved via a 5′-amino linker, the 3′-end was accessible to DNA polymerase. Addition of a (dT)_10_ sequence to the 5′-end of the primers yielded better hybridisation efficiency of the cDNA to the surface-bound molecules^14^. About 7.5 fmol of immobilised primer pairs in spots with a diameter of about 100 μm were optimal for successful amplification. The quality of array-bound DNA-products was examined by hybridisation of fluorescently labelled oligonucleotides that were complementary to the two newly synthesised DNA strands (Fig. 1b). Alternatively, primers were added to the immobilised DNA-templates. A polymerase reaction generated single-strand copies that were subsequently eluted from the microarray and amplified in solution. The resulting PCR-products were characterised by electrophoresis (Fig. 1b). From such quality control experiments, it was determined the intra- and inter-array coefficients of variation ranged between 5% and 20% up to a size of some 3 kb. For larger molecules, the yield varied strongly, although the polymerase used was suitable for synthesising long fragments.

The microarrays do not represent the proteins equivalent to mRNA abundance. On the contrary, for each protein a very similar amount of protein is present on the array, irrespective of the fact whether the respective mRNA is rare or frequent in the RNA isolate. By virtue of the on-chip polymerase elongation reaction and, independently from it, also due to the subsequent cell-free transcription and translation, the differences are mostly levelled. Consequently, similar amounts of protein will be produced at each microarray position. As a matter of fact, we have shown that usually the loading capacity of the microarray surface is limiting and thus determining the amount of protein present^11^.

### Protein quality

In order to define the efficiency of the *in situ* transcription and translation process, templates were used that had been produced with common primers from 2016 full-length cDNAs, which were randomly selected by using 21 microtiter plates of a library of in total about 12,000 non-redundant, full-length and sequence-verified clones^15^. Prokaryotic cell lysate was used for *in situ* protein expression. Using the MIST approach^11^, the reaction takes place in a small droplet at each spot. The droplets serve as separate compartments, thereby preventing cross-contamination. The newly expressed proteins are retained at the location of synthesis. As opposed to other approaches, there is no particular process of attachment to the surface, such as a chemical reaction or any means of specific affinity binding^7^,^9^. The proteins stick to the surface by non-covalent interaction with the epoxy-groups at the respective slide locations. In subsequent washing or incubation steps, no loss or movement of the *in situ* synthesised proteins could be detected. We prefer unspecific immobilisation, since the random orientation makes accessible all sides of a protein.

**Figure 2:**
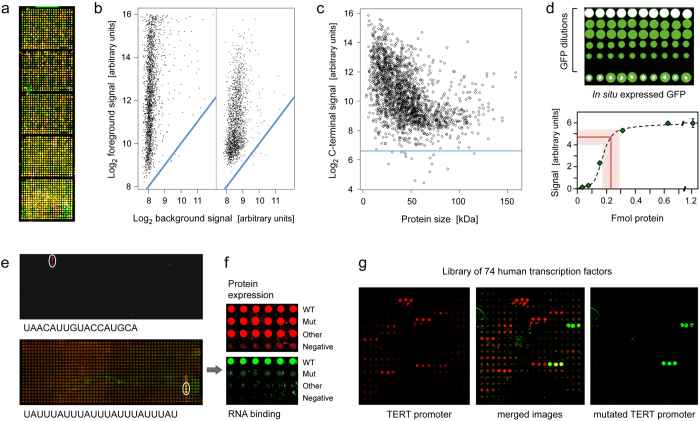
Protein microarray quality. (**a**) Microarray with 2016 proteins that were expressed *in situ*; visualisation was by incubation with red and green labelled antibodies that recognise common N- or C-terminal epitopes, respectively. (**b**) The typical ratio is shown of foreground to background signals for the N- (left) and C-terminus (right); blue lines indicate regions of identical intensity. (**c**) Detection of the C-termini of the 2016 expressed proteins with Cy3-conjugated anti-V5 antibody; the horizontal line represents a signal of three standard deviations above background. (**d**) Determination of the amount of *in situ* synthesised GFP by comparison to spotted material of known concentration; twenty measurements each were done; the red line represents the average amount of synthesised protein plus/minus one standard deviation. (**e**) Microarrays of *T. brucei* proteins were incubated with the labelled, synthetic RNA sequences shown. The white circles highlight positive signals. On the lower array, also a second protein exhibited interaction. In (**f**), the interacting protein from the lower microarray was analysed in more detail in comparison to a derivative with one point mutation (Mut) and another, unrelated protein. The upper panel shows the protein amounts, detected by antibody binding to the N-terminus. The lower panel shows binding of the synthetic RNA. Subsequent studies demonstrated a more than 3,000-fold difference in affinity to the specific RNA between wildtype and mutated protein. (**g**) Binding to 74 human transcription factors of a labelled synthetic DNA sequence representing the TERT promoter (left). Mutation of one base pair in the binding sequence led to a very different binding pattern (right).

Subsequent to protein expression, the arrays were incubated with fluorescently labelled antibodies that bound all proteins specifically at their N-terminal 6xHis or C-terminal V5 tags, respectively (Fig. 2a,b). From the signal intensities, it can be concluded that more than 90% of the protein spots contained full-length protein. For most proteins, 50% to 90% of the total protein amount was full-length, the actual yield being dependent on protein size (Fig. 2c). Some kind of limit was reached at about 110 kDa, equivalent to a length of the DNA template of about 3 kb. The reason for this is a combination of the restrictions of the initial polymerase reaction and the subsequent transcription and translation process. Nevertheless, some proteins could be expressed to full-length that were substantially larger. Also, the median of the mass of human proteins is about 42 kDa, substantially less than 110 kDa. Therefore, the majority of human proteins could be presented on the microarray.

A comparison of the signals generated by known amounts of spotted green fluorescence protein (GFP) with that of *in situ* synthesized molecules showed that around 0.2 fmol of protein was expressed on the array surface (Fig. 2d). From this experiment, it could also be concluded that only a small portion of the surface capacity is quenched by proteins that are present during the primer elongation reaction and the *in situ* translation process, which is not surprising given the relatively low protein concentration in the reactions. The capacity of about 0.2 fmol was confirmed by protein-staining with luminescence dye Sypro Ruby of some 14,000 different protein molecules expressed from PCR-products representing the genes of *Trypanosoma brucei* (Supplementary Fig. 2). The protein expression produced an average intra- and inter-array coefficient of variations of 14.6%, ranging from 7.2% to 21.6%. Also, as expected, most proteins were produced in very similar amounts (Supplementary Fig. 3). On purpose, we selected a few of the proteins that exhibited different yields in order to check reproducibility. Despite their differences in overall protein yield, reproducibility for each protein was nevertheless high. In addition, reproducibility was high both on microarrays produced as part of one production batch as well as on microarrays from different batches that were produced several weeks apart (Supplementary Fig. 4).

**Figure 3:**
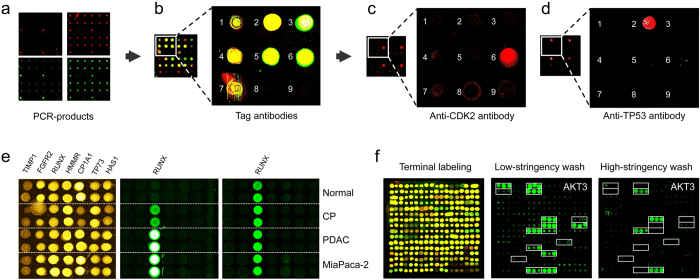
Detection of proteins generated *in situ* from individual samples. (**a**) Quality assessment of on-chip PCR by oligonucleotide hybridisation; two oligonucleotides were used, labelled red or green, respectively, each binding to one of the DNA-strands. Typical results are shown with an oligonucleotide specific to only one PCR-product present in quadruplicate (left) and a simultaneous hybridisation with oligonucleotides to all PCR-products (right). (**b**) Fusion of the images obtained after an incubation with fluorescently labelled antibodies against N- (red signal) and C-terminal tags (green signal) of the expressed seven tumour marker proteins. Spots 8 and 9 were negative controls without DNA-template. (**c**, **d**) Protein detection with labelled antibodies that target proteins CDK2 and TP53, respectively. (**e**) Results obtained on arrays produced from tissue samples of individual patients. Normal = healthy pancreas; CP = chronic pancreatitis; PDAC = pancreatic ductal adenocarcinoma; MiaPaca-2 = PDAC cell line. All proteins were identified with a tag-specific antibody (left). Binding patterns obtained with two different, isoform-specific antibodies. One isoform of the RUNX1 protein was present in all samples (right); the other one was found in diseased material only (middle). (**f**) Ninety-six DARPin binders were expressed *in situ*, each in three copies. Tag-specific antibodies identified all binders (left). The other two panels (middle, right) show binding patterns obtained upon incubation with protein AKT3. The white frames indicate the 16 binders that were expected to interact with AKT3. Different washing stringency produced distinct variations in the binding patterns.

The fluorescence of GFP also indicated that *in situ* synthesised protein could fold correctly and be functional. Given the very wide variety of protein size, structure and biochemistry, however, it is difficult to predict how many proteins may fold properly. We have performed a substantial number of incubations with antibodies, including few binders that are meant to identify structural epitopes. We have made the experience that some of the latter could also bind to linear epitopes, however. In experiments about the interaction of different molecule types, including protein-RNA interactions, protein-DNA interactions and protein-protein interactions (Figs 2e–g and 3f), we found many cases, in which proteins exhibited the expected functionality and specificity. However, the data is insufficient and probably also too biased to make from this a serious estimate about the overall percentage of properly folded proteins.

### Sample analysis

In addition to using oligomer hybridisation and tag-specific antibodies for quality control (Fig. 3a,b), *in situ* transcript amplification and subsequent protein expression were confirmed with antibodies that target individual proteins. Detection of binding was done with a secondary, fluorescently labelled antibody that was binding to the Fc part of the primary, protein-specific antibodies (Fig. 3c,d). In order to demonstrate the acquisition of personalised information, we studied tumour tissues of patients with pancreatic ductal adenocarcinoma – a tumour entity whose mortality is close to incidence and the forth most frequent cause of cancer-related death^16^ – in comparison to samples of matching healthy donors. mRNA preparations from tissues of either healthy donors, patients with chronic pancreatitis or cancer patients as well as a tumour cell line were copied onto microarrays and expressed. Antibodies were utilised that recognise different isoforms of the same protein. They belong to a set of antibodies that were made for identifying cancer-associated variations that are due to differential splicing (unpublished data). Distinct variations were detected between healthy and disease samples in the expression of RUNX1 (Fig. 3e), for example. By sequencing, the relevant splice variance, skipping of exon 6 in the *RUNX1* transcript, could be confirmed.

## Discussion

The procedure reported here enables an analysis of variations at the protein level that occur in individual samples. Basically, any number of proteins could be studied, although our work dealt with still relatively few molecules to date. In combination with the ability to synthesise oligonucleotides *in situ* on array surfaces in 5′ to 3′ direction in a directed and quantitative manner^17^, even each individual array could be different to the next for a truly targeted and individualised analysis. Nothing of this kind could be achieved at a reasonable cost and effort by individually purified proteins. The isolation of many distinct transcripts from each particular sample, cloning them individually, expressing them in *E. coli* or other expression systems, and purifying them prior to the eventual use on a microarray is simply far too work-intensive to be done for many samples.

The procedure yields proteins in the conformations that are encoded in the particular RNA-preparation used. This could lead to the presence of more than one protein at a particular spot, if the respective primer pair could bind to more than one transcript. Also, the quality of the RNA-preparation and subsequent reverse-transcription to cDNA is important. The initial step of on-chip polymerase elongation levels out most differences in abundance. The process does not produce microarrays that represent the abundance of particular RNAs in the isolate. Therefore, the RNA isolation does not have a real influence on the microarray. Still, methods for RNA isolation exist that are sufficient even for clinical applications. Also, analyses done on the microarray are of more qualitative and at best semi-quantitative nature. Some initial information about protein interaction stability could be obtained by different washing steps and repeated detection. For such studies, however, a control has to be added, such as labelling of the arrayed proteins via the terminal tag sequences, by which a normalisation is made possible for each spot on every microarray.

The experiments reported here, were performed with lysates from *E. coli*, as it was the most cost-effective process and yielded good results in terms of protein quality. However, we are aware of limitations that result from this. Therefore, we also studied lysates from rabbit, wheat, or *Drosophila*. They all worked efficiently, too. By this means, the effect of structural variations on posttranslational modifications could be investigated, for example. This could add yet another level of complexity and variance. In collaboration, we are currently studying a large variety of lysate types in order to identify the ones that are best tailored to different applications of the overall procedure. This should make the tool even more versatile.

The established process of on-chip PCR and subsequent protein expression provides the basis for a flexible and wide-ranging applicability for the analysis of samples that were isolated from particular tissues or individuals, or represent different states of *in vitro* processes, such as the selection of binders from recombinant libraries. The expression of single-chain antibody fragments or other affinity reagents, for example Designed Ankyrin Repeat Proteins^18^ (DARPins), could permit studies with respect to their specificity and affinity to target proteins and thus parallel selection of the best performing molecule from a library. Incubation of DARPins with their target proteins and others at different stringency conditions indicates the most specific binders (e.g., Fig. 3f). Such an approach could shorten significantly the process of binder identification and characterisation by combining the selection of suitable antibodies with the identification of biomedically informative target molecules^19^. This could be extended to presenting the variable domains of the antibodies that are most prominent in a patient by studying RNA isolated from circulating B-cells, for instance. Primer tag sequences already in use for multiplex sequencing could be used to such ends. Thereby, variations in many individual cells could be studied at both the sequence and protein level.

## Methods

### Oligonucleotides

All oligonucleotides were obtained from biomers.net (Ulm, Germany). Forward and reverse primer pairs were designed to amplify the respective open reading frame in full. Forward primers contained a ribosomal binding site (RBS), optionally a Kozak sequence, a T7 promoter, a 6× His epitope tag sequence, and a transcript-specific portion. Reverse primers were made of the V5 epitope sequence and a transcript-specific sequence; the presence of a T7 terminator sequence was found to be unnecessary. The Kozak sequence, which is important for the initiation of translation in eukaryotic cells, was not added, if prokaryotic cell lysates were used for protein expression. Primers that were attached to solid support had a (dT)_10_ stretch at their 5′-ends, which acted as a linker. Attachment to the epoxysilane-coated microarray surface occurred via an amino-group, which was chemically added during synthesis to the 5′-end as part of a C_6_-linker. The fluorescent Cy5- or Cy3-label of oligonucleotides used for hybridisation was added during chemical synthesis. The actual oligonucleotide sequences are provided as Supplementary Materials information.

### Primer attachment

Primer oligonucleotides were diluted to a concentration of 5 μM in 150 mM phosphate buffer, pH 8.5, 0.001% Tween 20. A volume of 1.5 nl was placed onto epoxysilane-coated slides (Nexterion E; Schott, Jena, Germany) using a NanoPlotter 2.0 non-contact piezo-system (Gesim, Großerkmannsdorf, Germany). Printed slides were kept at room temperature and 70% humidity for 24 h and stored dry for a minimum of two days before being used. Prior to the on-chip polymerase elongation reaction, the slides were blocked in superblock buffer (Thermo Fisher Scientific, Waltham, USA) at room temperature for 2 h. The slides were washed three times in distilled water and spin dried in a swing-wing centrifuge.

### RNA extraction and cDNA synthesis

Total RNA was extracted from the respective biological material (tumour, chronic pancreatitis and healthy pancreatic tissues as well as the pancreatic cancer cell line MiaPaca-2) using Trizol (Thermo Fisher Scientific) according to the manufacturer’s instructions. The RNA concentration was determined by absorption measurement on a Nanodrop ND-1000 (Peqlab Biotechnologie, Erlangen, Germany). The RNA was stored at −80 °C or used immediately for cDNA synthesis. cDNA was produced from 1 μg of total RNA by means of the Protoscript first-strand cDNA synthesis kit (New England Biolabs, Frankfurt, Germany) following the manufacturer’s protocol. One tenth of the product was used for the on-chip polymerase elongation reaction.

### On-chip polymerase elongation reaction

An adhesive gene frame (Thermo Fisher Scientific) was fixed onto the microarray and filled with 25 μl of 1x Qiagen LongRange PCR buffer, 2.5 mM MgCl_2_, 0.2 mM of each dNTP, 100 ng DNA and 1 U LongRange PCR polymerase (Qiagen, Hilden, Germany). Primer extension was done in a 16 × 16 dual-block PTC 200 thermocycler (BioRad, Munich, Germany) by initial heating to 93 °C for 3 min, followed by 35 cycles of 93 °C for 45 s, 50 °C for 45 s, 68 °C for 3 min, and a final elongation step at 68 °C for 10 min. The microarrays were extensively washed with water to remove the PCR cocktail and dried using pressurised air.

### Detection of on-chip DNA-products by oligonucleotide hybridisation

Fluorescently (Cy3 or Cy5) labelled oligonucleotides were diluted to 1 μM in 3× SSC (450 mM NaCl, 45 mM sodium citrate buffer, pH 7.0) supplemented with 0.1% SDS. After 10 min at 95 °C, hybridisation was at 60 °C overnight. The microarrays were subsequently washed in 3× SSC supplemented with 0.1% SDS, then in 3× SSC and finally in 0.3× SSC for 10 min each. The arrays were briefly rinsed with water and dried with pressurised air. The signal intensities were detected on a Power Scanner (Tecan, Männedorf, Switzerland).

### Detection of on-chip DNA-products by PCR in solution

The microarrays were extensively washed with water and dried. Adhesive gene frames (Thermo Fisher Scientific) were placed on the microarrays and filled in with 1x Qiagen PCR buffer, 2.5 mM MgCl_2_, 0.2 mM of each dNTP, 0.2 μM of each primer, and 1 unit of Taq polymerase (Qiagen). However, no DNA template was added but for the DNA present on the microarray spots. The amplification reaction was performed as described for the on-chip polymerase elongation reaction. The PCR-products in the supernatant were subjected to agarose gel electrophoresis. As a negative control, spots were used on which there was no DNA-template.

### *In situ* cell-free protein expression

For protein expression, slides with PCR-products were washed twice in 1xSSC for 5 min and dried. Multiple spotting (MIST) was used for *in situ* cell-free expression as described in detail before^11^. In short, the slides were placed into the NanoPlotter 2.0 non-contact piezo-element system (Gesim) in the same orientation they had been during primer spotting. First, 0.6 nl of 0.5 M betaine was spotted onto the position of each PCR-product, followed by 2.1 nl of S30 T7 High-Yield Protein Expression System (Promega, Madison, USA). In a humidified chamber, the slides were incubated at 37 °C for 1 h and subsequently at 30 °C overnight. After washing, the microarrays were stored at −20 °C for a minimum of 24 h before use. Usually, we use a density of about 2,000 proteins per slide of 7 × 2 cm. At higher densities, there is a higher risk that the individual droplets, in which the reactions take place, may get in contact, thus causing contamination. However, spot density can be adapted to the respective requirements.

### Protein detection

#### Sypro Ruby staining

For a quick determination of overall array quality, protein microarrays were incubated with Sypro Ruby staining solution (Thermo Fisher Scientific). The resulting luminescence signal is equivalent to the amount of protein present at each location.

#### Antibody detection

For a more detailed analysis, expressed proteins were detected with directly fluorescence-labelled antibodies that recognise the N-terminal 6xHis epitope (Penta-His Alexa Fluor 647 Conjugate; Qiagen) or the C-terminal V5 epitope (monoclonal anti-V5-Cy3; Sigma-Aldrich, St. Louis, USA). In addition, protein-specific monoclonal antibodies were used (e.g., anti-CDK2, clone AN4.3 and anti-P53, clone DO-1; Sigma-Aldrich). The microarrays were blocked with 2% BSA in phosphate buffered saline (137 mM sodium chloride, 2.7 mM potassium chloride, 10.0 mM disodium hydrogen phosphate, 1.76 mM sodium dihydrogen phosphate, pH 7.4) supplemented with 0.05% Tween 20 (PBST) for 30 min. Blocking with 2% BSA was required so as to cover the regions in between protein spots, since otherwise the labelled antibodies would bind preferentially to these areas. There was very little unspecific binding to the actual protein spots even without blocking, but the blocking does not harm specific binding events. After washing the arrays surface in PBST three times, antibody incubation was in PBST, 2% BSA at room temperature on a rotary shaker for 1 h. While 0.1 μg/ml binder was used for epitope tag detection, protein-specific antibodies had a final concentration of 0.5 μg/ml. For detection of the latter, the microarrays were washed three times in PBST for 10 min and incubated with a 1:10.000 dilution of fluorescent dye conjugated secondary antibody (Goat Anti-Mouse IgG Cy5-conjugated antibody; Jackson ImmunoResearch, Newmarket, UK) for another hour. Finally, the microarrays were washed three times in PBST for 10 min and air-dried in a ventilated oven.

#### DARPin targets

Target proteins were enzymatically biotinylated using the ImmunoProbe Biotinylation kit (Sigma-Aldrich). After incubation of 5 μg/ml biotinylated protein on the DARPin array, detection was performed with a 1:500 dilution of Cy3-Etravidin (Sigma-Aldrich) for 1 h.

### Data acquisition and analysis

For signal detection, microarrays were scanned on a Power Scanner (Tecan, Männedorf, Switzerland). In all measurements, the instrument’s laser power and photomultiplier tube were adjusted to avoid signal saturation. The scanner settings were kept identical for all arrays of an experimental series. Relevant parameters, such as concordance of the two colour detection channels, were carefully validated throughout. Spot segmentation was performed with GenePix Pro 6.0 (Molecular Devices, Union City, USA). The mean local background intensity was subtracted from the mean signal intensity for each spot to obtain background-corrected signal intensities. Data were analysed using the linear models for microarray data (LIMMA) package^20^ of R-Bioconductor. For normalisation, a specialised invariant Lowess method was applied^21^.

## Additional Information

**How to cite this article**: Syafrizayanti, *et al*. Personalised proteome analysis by means of protein microarrays made from individual patient samples. *Sci. Rep.* **7**, 39756; doi: 10.1038/srep39756 (2017).

**Publisher's note:** Springer Nature remains neutral with regard to jurisdictional claims in published maps and institutional affiliations.

## Supplementary Material

Supplementary Material
