# Supplementary material for: Personalised proteome analysis by means of protein microarrays made from individual patient samples
Source: Sci Rep. 2017 Jan 3;7:39756. doi: 10.1038/srep39756 (PMC5206632; doi:10.1038/srep39756)
Supplement: Supplementary Material [file srep39756-s1.pdf]

Personalised proteome analysis by means of protein microarrays made from individual patient samples

Syafrizayanti, Smiths S. Lueong, Cuixia Di, Jonas V. Schäfer, Andreas Plückthun
and Jörg D. Hoheisel

1. **Supplementary Figures:** page 1 to 3
2. **Supplementary Material:** oligonucleotide sequences; pages 4 to 9

Supplementary Figures

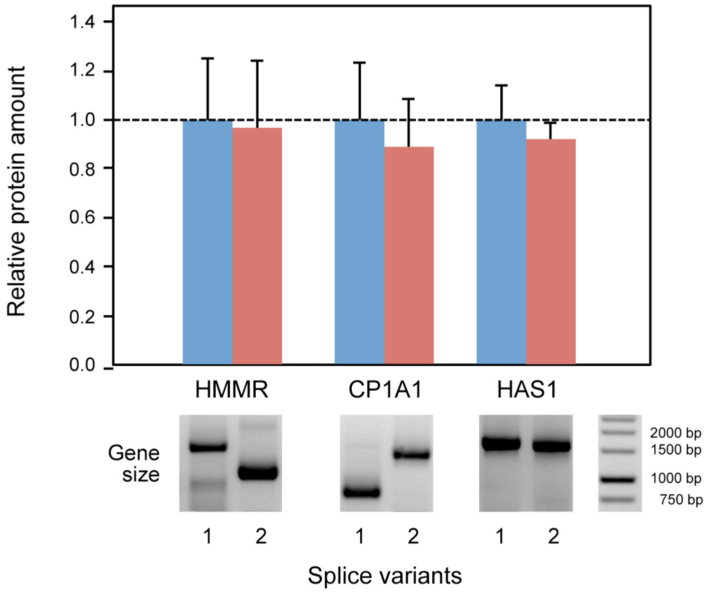

Supplementary Figure 1. Isoform representation on the protein microarray. Two splice variants each of three genes were expressed on the protein microarray, starting with the same amount of each variant. Irrespective of the actual size difference between the gene isoforms (shown at the bottom), similar amounts of protein were produced on the microarray surface.

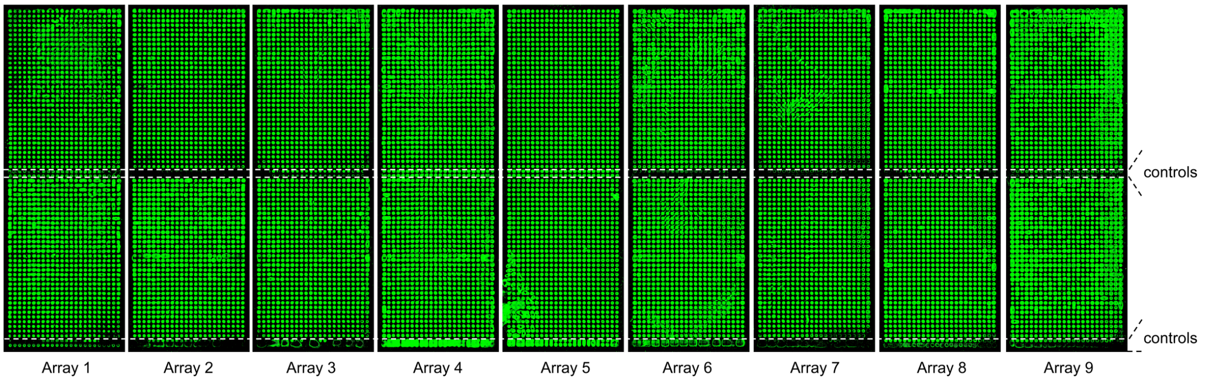

Supplementary Figure 2. Measurement of protein content by luminescent labelling. Nine protein microarrays were produced containing proteins expressed from about 5 pg each of some 14,000 different PCR-products representing *T. brucei* genes. The microarray-bound proteins were labelled with the luminescent dye Sypro Ruby for quality control purposes.

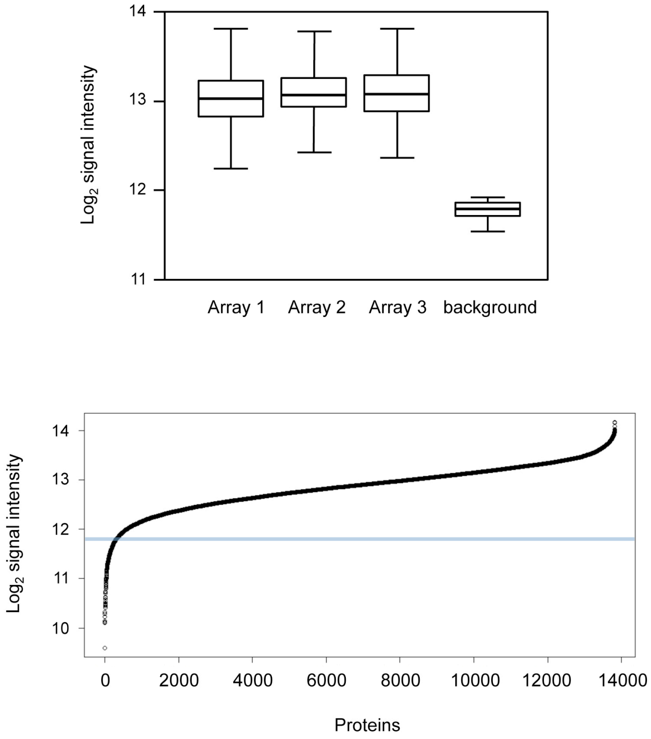

Supplementary Figure 3. Microarray reproducibility. Top panel: Box plot of the overall signal variations after Sypro Ruby labelling of three randomly selected microarrays containing 1,560 proteins each. In the bottom panel, the variation in signal intensity is shown for about 14,000 *T. brucei* proteins produced on microarrays. The blue horizontal line indicates the intensity of the background signal.

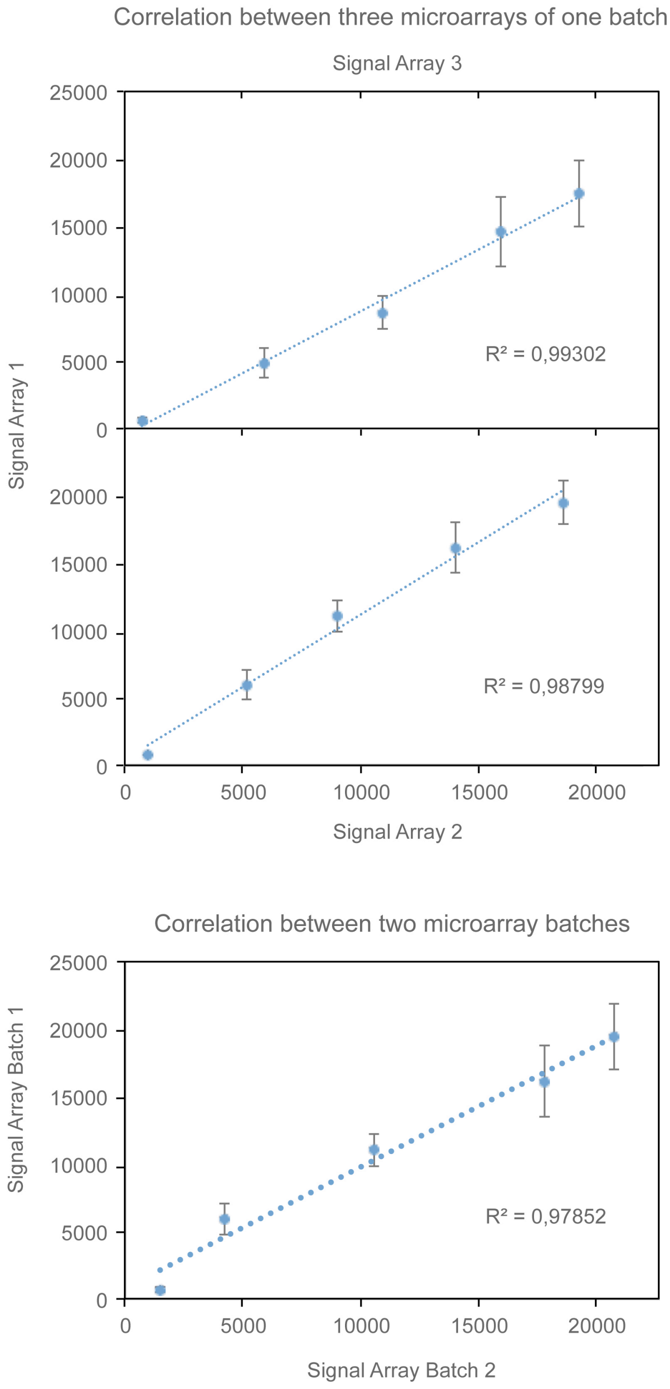

Supplementary Figure 4. Microarray reproducibility. Top: Correlation of protein expression on different microarrays produced in a single batch is shown for FGFR, HMMR, TPM and CP1A1. The median of 10 measurements is given. The left-bottom data point in each panel indicates the negative control. Bottom: The same kind of data is provided for microarrays that were produced as part of different microarray batches.

Supplementary Material: oligonucleotide sequences

In some initial primer constructs for the generation of DNA templates for *in situ* protein production, we had included a T7 terminator sequence to stop transcription. However, this was found to be unnecessary, since transcription stopped at the end of each DNA template anyway. Also the Kozak sequence, which plays a major role in the initiation of the translation process in eukaryotic cells, was not added to primer sequences, if prokaryotic extracts were used for cell-free protein expression.

Primers that were attached to the solid support had a $(dT)_{10}$ stretch present at their 5'-ends, which acted as a linker. Attachment to the microarray occurred via an amino-group, which was chemically added during synthesis to the 5'-end as part of a C_6-linker.

Below, the oligonucleotide sequences are listed, which were used in the work described in the manuscript. Sequence sections that are colored are of functional or technical relevance.

Common Gateway primers for DNA template construction by PCR on individual cDNA clones in Gateway vector pDONR221.

Name	5′ - 3′
Fw_ Gateway_com	TCCCGCGAAATTAATACGACTCACTATAGGGAGACCACAACGGTTTCCCTCTAGAAATAATTAGCCACCATGGTAAGAAGGAGATATACCATGCATCATCATCATCATAAAGCAGGCTCCACCATG
Rev_ Gateway_com	CTGGAATTCGCCCTTTTATTACGTAGAATCGAGACCGAGGAGAGGGTTAGGGATAGGCTTACCAACTTTGTACAAGAAAGCTGGGTC

- T7 promoter; Kozak sequence; ribosomal binding site (RBS)
- Sequences encoding epitope tags: 6×His and V5
- Underlined sequences are binding sites of the *common solid-phase primers*
- Blue sequences act as common primers in Gateway vector pDONR221

Color-coded representation of the DNA templates created with the *Common Gateway primers*

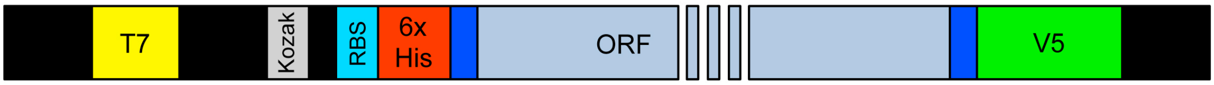

ORF: open reading frame

Common solid-phase primers for on-chip re-amplification of cDNA-derived DNA templates

Name	5′ - 3′
SPh-Fw_com	NH_3-C6-TTTTTTTTTTAGGTGCGTGTGGGTTGGATCTCCCGCGAAATTAATACG
SPh-Rev_com	NH_3-C6-TTTTTTTTTTGATTAGAAAGTAACCTCAGTCTGGAATTCGCCCTTTTATTA

The underlined sequences recognize the 5′-end sequences of the respective *Common Gateway primers*.

In-solution PCR primers for determining DNA-sizes from array-bound PCR-products

Name	5′ - 3′
SPh-Fw_com-terminal	TCCCGCGAAATTAATACG
SPh-Rev_com-terminal	CTGGAATTCGCCCTTTTATTA

Gene-specific primers for DNA template generation

- T7 promoter; ribosomal binding site (RBS)
- Sequences encoding epitope tags: 6×His and V5
- Blue sequences are gene-specific sequences
- Orange, underlined sequences are unique sequences for re-amplification

Name	5′ - 3′
Fw-U1_DIABLO	TCCATGGAACCGTACTCGCCGTGTAATACGACTCACTATAGGGAGACCACAACGGTTTCCCTCTAGAAATAATTAGGAGATATACCTAATGCATCATCATCATCATCATTTGTGTGTTCCTGTTGTGGC
Rev-U1_DIABLO	CTGGCGTAGAGTCGCATCCAGTCTTATTACGTAGAATCGAGACCGAGGAGAGGGTTAGGGATAGGCTTACCTTTCTGACGGAGCTCTTCTATC
Fw-U2_CFLAR	TCCTACCCTCGTGGTTGCGCTAATAATACGACTCACTATAGGGAGACCACAACGGTTTCCCTCTAGAAATAATTAGGAGATATACCTAATGCATCATCATCATCATCATTCTGCTGAAGTCATCCATCAG
Rev-U2_CFLAR	CTGTAGACCCTGTCGGCTACGGATTATTACGTAGAATCGAGACCGAGGAGAGGGTTAGGGATAGGCTTACCGTGCTGCAGCCAGACATAATA
Fw-U3_CFLAR-N18aa	TCCGCGTCTTATACGGAACCGCGTAATACGACTCACTATAGGGAGACCACAACGGTTTCCCTCTAGAAATAATTAGGAGATATACCTAATGCATCATCATCATCATCATGAGATGCTGTCTTTTTGTGC
Rev-U3_CFLAR-N18aa	CTGTATCGTAGGGCCACTCAGGCTTATTACGTAGAATCGAGACCGAGGAGAGGGTTAGGGATAGGCTTACCGTGCTGCAGCCAGACATAATA
Fw-U4_AKT1	TCCGAACATTGGACTGGCACGGGTAATACGACTCACTATAGGGAGACCACAACGGTTTCCCTCTAGAAATAATTAGGAGATATACCTAATGCATCATCATCATCATCATGCTCCCCTCAACAACTTCTC
Rev-U4_AKT1	CTGTTGGTCCGATGCCACTTGCGTTATTACGTAGAATCGAGACCGAGGAGAGGGTTAGGGATAGGCTTACCGTCATCTTGGTCAGGTGGTGT
Fw-U5_MAPK1	TCCGACCTTGGCAGTCGCTAACGTAATACGACTCACTATAGGGAGACCACAACGGTTTCCCTCTAGAAATAATTAGGAGATATACCTAATGCATCATCATCATCATCATGAGATGGTCCGCGGGCAGG

Rev-U5_MAPK1	CTGGGGATGAACGGCTATCGCAGTTATTACGTAGAATCGAGACCGAGGAGAGGGTTAGGGATAGGCTTACCGGGCTCGTCACTCGGGTCGTAATA
Fw-U6_BCL2L1	TCCCGATCTGGGAGGTATGGCTTTAATACGACTCACTATAGGGAGACCACAACGGTTTCCCTCTAGAAATAATTAGGAGATATACCTAATGCATCATCATCATCATCATTCTCAGAGCAACCGGGAGCT
Rev-U6_BCL2L1	CTGCCCATCGGAATCGAGTGTGGTTATTACGTAGAATCGAGACCGAGGAGAGGGTTAGGGATAGGCTTACCCCGACTGAAGAGTGAGCCCAG
Fw-U7_CDK2	TCCCAGTCAACACTGGAGGCTCGTAATACGACTCACTATAGGGAGACCACAACGGTTTCCCTCTAGAAATAATTAGGAGATATACCTAATGCATCATCATCATCATCATAGAAACAAGTTGACGGGAGAG
Rev-U7_CDK2	CTGAGTGAGACGCTCGAGTAGCATTATTACGTAGAATCGAGACCGAGGAGAGGGTTAGGGATAGGCTTACCATGGGGTACTGGCTTGGTC
Fw-U1_TIMP1	ATGGAACCGTACTCGCCGTGGAAATTAATACGACTCACTATAGGGAGACCACAACGGTTTCCCTCTAGAAATAATTTTGTTTAAGAAGGAGATATACATATGCATCATCATCATCATCATATGGACGCCATCAAGA
Rev-U1_TIMP1	GCGTAGAGTCGCATCCAGTCCTGGAATTCGCCCTTTTATTACGTAGAATCGAGACCGAGGAGAGGGTTAGGGATAGGCTTACCCATGGAAGTCATATCGTTG
Fw-U2_FGFR2	TACCCTCGTGGTTGCGCTAAGAAATTAATACGACTCACTATAGGGAGACCACAACGGTTTCCCTCTAGAAATAATTTTGTTTAAGAAGGAGATATACATATGCATCATCATCATCATCATATGGTCAGCTGGGGTC
Rev-U2_FGFR2	TAGACCCTGTCGGCTACGGACTGGAATTCGCCCTTTTATTACGTAGAATCGAGACCGAGGAGAGGGTTAGGGATAGGCTTACCTGTTTTAACACTGCCGTT
Fw-U3_CP1A1	GCGTCTTATACGGAACCGCGGAAATTAATACGACTCACTATAGGGAGACCACAACGGTTTCCCTCTAGAAATAATTTTGTTTAAGAAGGAGATATACATATGCATCATCATCATCATCATATGCTTTTCCCAATCTCC
Rev-U3_CP1A1	TATCGTAGGGCCACTCAGGCCTGGAATTCGCCCTTTTATTACGTAGAATCGAGACCGAGGAGAGGGTTAGGGATAGGCTTACCAGAGCGCAGCTGCAT
Fw-U4-HMMR	GAACATTGGACTGGCACGGGGAAATTAATACGACTCACTATAGGGAGACCACAACGGTTTCCCTCTAGAAATAATTTTGTTTAAGAAGGAGATATACATATGCATCATCATCATCATCATATGTCCTTTCCTAAGGCG
Rev-U4-HMMR	TTGGTCCGATGCCACTTGCGCTGGAATTCGCCCTTTTATTACGTAGAATCGAGACCGAGGAGAGGGTTAGGGATAGGCTTACCCTTCCATGATTCTTGACACTC
Fw-U5-RUNX	GACCTTGGCAGTCGCTAACGGAAATTAATACGACTCACTATAGGGAGACCACAACGGTTTCCCTCTAGAAATAATTTTGTTTAAGAAGGAGATATACATATGCATCATCATCATCATCATATGGCTTCAGACAGCATAT
Rev-U5-RUNX	GGGATGAACGGCTATCGCAGCTGGAATTCGCCCTTTTATTACGTAGAATCGAGACCGAGGAGAGGGTTAGGGATAGGCTTACCGTAGGGCCTCCACACG
Fw-U6-TP73	CGATCTGGGAGGTATGGCTTGAAATTAATACGACTCACTATAGGGAGACCACAACGGTTTCCCTCTAGAAATAATTTTGTTTAAGAAGGAGATATACATATGCATCATCATCATCATCATATGCTGTACGTCGGTGA
Rev-U6-TP73	CCCATCGGAATCGAGTGTGGCTGGAATTCGCCCTTTTATTACGTAGAATCGAGACCGAGGAGAGGGTTAGGGATAGGCTTACCGTGGATCTCGGCCTC
Fw-U7-HAS1	CAGTCAACACTGGAGGCTCGGAAATTAATACGACTCACTATAGGGAGACCACAACGGTTTCCCTCTAGAAATAATTTTGTTTAAGAAGGAGATATACATATGCATCATCATCATCATCATATGGTCTGTGACTCGGAC
Rev-U7-HAS1	AGTGAGACGCTCGAGTAGCACTGGAATTCGCCCTTTTATTACGTAGAATCGAGACCGAGGAGAGGGTTAGGGATAGGCTTACCCACCTGGACGCGGTA

Color-coded representation of the DNA template created with the *gene-specific primers*

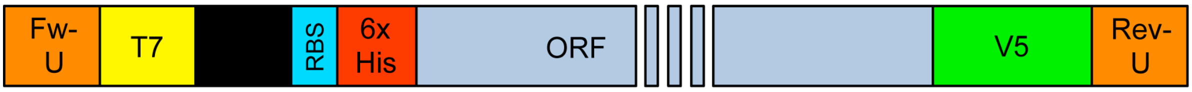

ORF: open reading frame
Fw-U, Rev-U: unique primer sequences for re-amplification

Unique solid-phase primers for on-chip re-amplification of gene-specific DNA templates

Name	5′ - 3′
SPh-Fw_U1	NH_3-C6-TTTTTTTTTTAGATGCATGTGAGTTAGATCATGGAACCGTACTCGCCGTG
SPh-Rev_U1	NH_3-C6-TTTTTTTTTTGATTAGAAAGTAACCTCAGTGCGTAGAGTCGCATCCAGTC
SPh-Fw_U2	NH_3-C6-TTTTTTTTTTAGATGCATGTGAGTTAGATCTACCCTCGTGGTTGCGCTAA
SPh-Rev_U2	NH_3-C6-TTTTTTTTTTGATTAGAAAGTAACCTCAGTTAGACCCTGTCGGCTACGGA
SPh-Fw_U3	NH_3-C6-TTTTTTTTTTAGGTGCGTGTGGGTTGGATCGCGTCTTATACGGAACCGCG
SPh-Rev_U3	NH_3-C6-TTTTTTTTTTGATTAGAAAGTAACCTCAGTTATCGTAGGGCCACTCAGGC
SPh-Fw_U4	NH_3-C6-TTTTTTTTTTAGATGCATGTGAGTTAGATCGAACATTGGACTGGCACGGG
SPh-Rev_U4	NH_3-C6-TTTTTTTTTTGATTAGAAAGTAACCTCAGTTTGGTCCGATGCCACTTGCG
SPh-Fw_U5	NH_3-C6-TTTTTTTTTTAGATGCATGTGAGTTAGATCGACCTTGGCAGTCGCTAACG
SPh-Rev_U5	NH_3-C6-TTTTTTTTTTGATTAGAAAGTAACCTCAGTGGGATGAACGGCTATCGCAG
SPh-Fw_U6	NH_3-C6-TTTTTTTTTTAGATGCATGTGAGTTAGATCCGATCTGGGAGGTATGGCTT
SPh-Rev_U6	NH_3-C6-TTTTTTTTTTGATTAGAAAGTAACCTCAGTCCCATCGGAATCGAGTGTGG
SPh-Fw_U7	NH_3-C6-TTTTTTTTTTAGATGCATGTGAGTTAGATCCAGTCAACACTGGAGGCTCG
SPh-Rev_U7	NH_3-C6-TTTTTTTTTTGATTAGAAAGTAACCTCAGTAGTGAGACGCTCGAGTAGCA

The orange underlined sequences recognize a 5′-sequence of *gene-specific primers*.
"NH_3-C6" stands for the amino group attached to the 5' end via a C6 linker.

Gene-specific primers with a NH_3-C6-(dT_{10}) linker at their 5' end.

- T7 promoter; ribosomal binding site (RBS)
- Sequence encoding epitope tag: V5
- Blue sequences are gene-specific sequences

Name	5′ - 3′
Fw-BCL2L1	NH_3-C6-TTTTTTTTTTGCATGTGAGTGAAATTAATACGACTCACTATAGGGAGACCACAACGGTTTCCCTCTAGAAATAATTTTGTTTAAGAAGGAGATATACATATGTCTCAGAGCAACCGGGAGCT
Rev-BCL2L1	NH_3-C6-TTTTTTTTTTGATTAGAAAGGTCTTATTACGTAGAATCGAGACCGAGGAGAGGGTTAGGGATAGGCTTACCCCGACTGAAGAGTGAGCCCAG

Fw-CDK2	NH_3-C6-TTTTTTTTTTGCATGTGAGTGAAATTAATACGACTCACTATAGGGAGACCACAACGGTTTCCCTCTAGAAATAATTTTGTTTAAGAAGGAGATATACATATGAGAAACAAGTTGACGGGAGAG
Rev-CDK2	NH_3-C6-TTTTTTTTTTGATTAGAAAGGTCTTATTACGTAGAATCGAGACCGAGGAGAGGGTTAGGGATAGGCTTACCATGGGGTACTGGCTTGGTC
Fw-CP1A1	NH_3-C6-TTTTTTTTTTGCATGTGAGTGAAATTAATACGACTCACTATAGGGAGACCACAACGGTTTCCCTCTAGAAATAATTTTGTTTAAGAAGGAGATATACATATGCTTTTCCCAATCTCC
Rev-CP1A1	NH_3-C6-TTTTTTTTTTGATTAGAAAGGTCTTATTACGTAGAATCGAGACCGAGGAGAGGGTTAGGGATAGGCTTACCAGAGCGCAGCTGCAT
Fw-FGFR2	NH_3-C6-TTTTTTTTTTGCATGTGAGTGAAATTAATACGACTCACTATAGGGAGACCACAACGGTTTCCCTCTAGAAATAATTTTGTTTAAGAAGGAGATATACATATGGTCAGCTGGGGTC
Rev-FGFR2	NH_3-C6-TTTTTTTTTTGATTAGAAAGGTCTTATTACGTAGAATCGAGACCGAGGAGAGGGTTAGGGATAGGCTTACCTGTTTTAACACTGCCGTT
Fw-HAS1	NH_3-C6-TTTTTTTTTTGCATGTGAGTGAAATTAATACGACTCACTATAGGGAGACCACAACGGTTTCCCTCTAGAAATAATTTTGTTTAAGAAGGAGATATACATATGGTCTGTGACTCGGAC
Rev-HAS1	NH_3-C6-TTTTTTTTTTGATTAGAAAGGTCTTATTACGTAGAATCGAGACCGAGGAGAGGGTTAGGGATAGGCTTACCCACCTGGACGCGGTA
Fw-HMMR	NH_3-C6-TTTTTTTTTTGCATGTGAGTGAAATTAATACGACTCACTATAGGGAGACCACAACGGTTTCCCTCTAGAAATAATTTTGTTTAAGAAGGAGATATACATATGTCCTTTCCTAAGGCG
Rev-HMMR	NH_3-C6-TTTTTTTTTTGATTAGAAAGGTCTTATTACGTAGAATCGAGACCGAGGAGAGGGTTAGGGATAGGCTTACCCTTCCATGATTCTTGACACTC
Fw-RUNX	NH_3-C6-TTTTTTTTTTGCATGTGAGTGAAATTAATACGACTCACTATAGGGAGACCACAACGGTTTCCCTCTAGAAATAATTTTGTTTAAGAAGGAGATATACATATGGCTTCAGACAGCATAT
Rev-RUNX	NH_3-C6-TTTTTTTTTTGATTAGAAAGGTCTTATTACGTAGAATCGAGACCGAGGAGAGGGTTAGGGATAGGCTTACCGTAGGGCCTCCACACG
Fw-TIMP1	NH_3-C6-TTTTTTTTTTGCATGTGAGTGAAATTAATACGACTCACTATAGGGAGACCACAACGGTTTCCCTCTAGAAATAATTTTGTTTAAGAAGGAGATATACATATGGACGCCATCAAGA
Rev-TIMP1	NH_3-C6-TTTTTTTTTTGATTAGAAAGGTCTTATTACGTAGAATCGAGACCGAGGAGAGGGTTAGGGATAGGCTTACCCATGGAAGTCATATCGTTG
Fw-TP73	NH_3-C6-TTTTTTTTTTGCATGTGAGTGAAATTAATACGACTCACTATAGGGAGACCACAACGGTTTCCCTCTAGAAATAATTTTGTTTAAGAAGGAGATATACATATGCTGTACGTCGGTGA
Rev-TP73	NH_3-C6-TTTTTTTTTTGATTAGAAAGGTCTTATTACGTAGAATCGAGACCGAGGAGAGGGTTAGGGATAGGCTTACCGTGGATCTCGGCCTC

Primers for amplification of DARPin binders.

- T7 promoter; ribosomal binding site (RBS)
- Sequences encoding epitope tags: 6×His and V5
- Blue sequences are gene-specific sequences
- "NH_3-C6" stands for the amino group attached to the 5' end via a C6 linker.

Name	5′ - 3′
Fw-DARPin	NH_3-C6-TTTTTTTTTTGCATGTGAGTGAAATTAATACGACTCACTATAGGGAGACCACAACGGTTTCCCTCTAGAAATAATTTTGTTTAAGAAGGAGATATACATATGCATCATCATCATCATCATATGAGAGGATCGCATCACCATC
Rev-DARPin	NH_3-C6-TTTTTTTTTTCTGGAATTCGCCCTTTTATTACGTAGAATCGAGACCGAGGAGAGGGTTAGGGATAGGCTTACCAGCTTTCTGAAGAACTTCAGCG

Gene-specific hybridisation oligonucleotides for the detection of on-chip DNA templates: nprb_fw-NAME oligonucleotides bind to extension products of the respective forward primers; nprb_rev-NAME oligomers bind to extension products of the reverse primers.

Name	5′ - 3′	Label
nprb_fw-CHMP2A	GCTTGTGCCATCGAGTTGTTGGACTTGAG	Cy3
nprb_rev-CHMP2A	GCTGTTCGCATCATGGCAAAAGACTTGGT	Cy5
nprb_fw-CREB3L1	GAGGGAGCCCAGCACCAGAACAAAG	Cy3
nprb_rev-CREB3L1	TTGATGACCCTGTGCTGGATGAGAAGAG	Cy5
nprb_fw-DIABLO	TATAGAGGCCTGATCTGCGCCAGTTTGATA	Cy3
nprb_rev-DIABLO	CTTGGGAAAATGAATTCAGAGGAGGAAGA	Cy5
nprb_fw-ICAM3	GCCCTTGTACTTGATGATGTGCTCGTGG	Cy3
nprb_rev-ICAM3	CTTTCTGCACCTCTGTCACCTCGCTCTCC	Cy5
nprb_fw-IDH3B	GGGCATCACAAGCACATCAAACTGGTAAGG	Cy3
nprb_rev-IDH3B	GTCAAGGAGGTGTTCAAGGCTGCCGCTGTC	Cy5
nprb_fw-IL1RN	AGCGCTTGTCCTGCTTTCTGTTCTCG	Cy3
nprb_rev-IL1RN	CTTGGGAATCCATGGAGGGAAGATGTGC	Cy5
nprb_fw-SORT1	TGTAGCCCAAAATGCAGCCATCTTCATAATCT	Cy3
nprb_rev-SORT1	GTTTGGCCAAATGGGGATCAGACAACA	Cy5
